# Supplementary material for: Are carbon emissions trading and green financial instruments synergistic? -Comprehensive quantitative research based on content analysis
Source: PLoS One. 2024 Mar 7;19(3):e0298601. doi: 10.1371/journal.pone.0298601 (PMC10919601; doi:10.1371/journal.pone.0298601)
Supplement: S1 Data — (ZIP) [file pone.0298601.s001.zip › Data/地级市Z_F工作报告.zip!/╝¬┴╓/░╫│╟╩╨/└┤╫╘╣1⁄2╓┌║┼í╛┬φ┐╦╩2╛▌═°í┐_2008.docx]

各位代表：

　　白城市第三届人民政府任期已四年，按照中央和省委的要求提前一年换届。现在，我代表白城市人民政府，向市第四届人民代表大会第一次会议作政府工作报告，请予审议，并请市政协各位委员提出意见。

　　一、过去四年的工作

2004年以来，在市委的正确领导下，在市人大依法监督和市政协的民主监督下，市政府团结带领全市广大干部群众，拼搏进取，扎实工作，经济社会实现了快速健康发展。

　　——综合经济实力大幅度提升。2007年，全市地区生产总值完成228亿元，比2003年增长102.3%，年均增长19.3%（预计数，按可比口径计算，下同）。固定资产投资完成200亿元，比2003年增长315.8%，年均增长42.8%。全口径财政收入17.1亿元，比2003年增长74.5%，年均增长14.9%。三次产业比重由2003年的36：28：36调整为今年的24：39：37，经济结构进一步优化。

　　工业速度和效益持续增长。2007年全市规模以上工业增加值完成28亿元，年均增长15.6%；实现利润3亿元，年均增长10.7%。四年完成技改265项，开发新产品310种。“绿原”牌纯棉坯布、“好雨”牌大米被评为中国名牌产品。县域工业快速发展，工业总产值年均增长36.8%。

　　农村经济实现新发展。2007年全市农业总产值完成88.2亿元。特色作物四年增加200万亩左右，白城燕麦、白城绿豆被评为地理标志保护产品。农产品加工企业发展到841户，牧业园区发展到160个。实施了引嫩入白、人畜饮水等工程。桑树、山杏、白刺面积分别达到52.3万亩、95万亩和26万亩。四年农村劳务输出143.8万人（次），创收428亿元，新农村建设有序推进。

　　商贸经济繁荣活跃。2007年全市社会消费品零售总额完成92.5亿元，年均增长16.6%。外贸进出口额完成6000万美元，年均增长355%，四年直接利用外资2810万美元，年均增长23.9%。

　　——工业园区和项目建设快速推进。建设了启动区22平方公里的白城工业园区，完成了11平方公里的“七通一平”，累计入园项目77个，总投资110亿元，其中在建39个，投产26个，一个蓬勃发展的工业新城正在迅速崛起。各县（市）工业园区全面启动，基础设施和项目建设均实现较快发展。

　　加快项目建设，滚动实施“百项工程攻坚”，全市四年实施千万元以上项目386个，完成投资245亿元。一是运作多年的十大重点项目取得突破性进展。白城电厂2×60万千瓦火电新建、白城热电厂扩建工程已获国家发改委批复性文件，火电新建工程已奠基；500千伏输变电工程已完成可研审核；引嫩入白工程在国家立项，白沙滩枢纽提水泵站和54公里总干渠已竣工；镇赉土地整理开发项目全面推进，大安土地整理开发项目正在做开工准备；大安灌区建设有序进行；白城机场已列入国家“十一五”民航机场规划；人工降雨基地二期项目正在做前期工作。二是风电项目建设全面铺开。全市立项风电场10个，建成了通榆同发、洮南大通、洮北华能和富裕、大安大岗子、镇赉青山、查干浩特等风电场，并网发电56万千瓦。三是农产品加工项目快速推进。洮北庭峰20万吨酒精一期已投产；公益源有机黑大豆综合加工、尼龙1010、洮南爱迪尔辣椒深加工项目已试生产；洮北哈工大日处理500吨鲜奶、燕麦系列饮品项目完成了阶段任务。四是工业扩能改造项目取得较好进展。华金纸业治污、大安石油机械技改、新天成水泥、金事达欧Ⅲ线束等项目陆续投产；金升镍业、双华水泥项目全面启动。

　　——改革开放实现新突破。完成了列入省计划的68户国有工业企业改革攻坚任务。积极实施民营经济腾飞计划，民营经济实现新发展。着力推进农村综合配套、事业单位人事制度和粮食流通体制等改革，发展活力不断增强。

　　大力实施招商引资。组织开展了振兴东北老工业基地“中国光彩事业白城行”等活动，四年招商引资到位401.5亿元，是前四年总和的3.3倍。开发区经济不断壮大，对外开放和招商窗口作用进一步增强。

　　积极发展特色旅游业。加强旅游基础设施建设，向海和莫莫格被评为国家4A级景区。围绕向海湿地特种邮票发行等，组织开展了一系列旅游活动，四年接待域外游客338万人（次），创收18亿元。

　　——城乡面貌发生显著变化。加快城乡基础设施建设，大力实施民生民利工程，市区四年完成投资84亿元，是前四年总和的2.1倍。棚户区改造基本实现了当年拆迁、当年建设、当年回迁目标。建设了运河公园、东部供热站、自来水厂、垃圾处理场，实施了市民广场和供热管网改造等。四年新建、改造道路120万平方米；新建住宅200万平方米，市区人均居住面积达到22.9平方米；建成区绿化覆盖率达36.5%，空气质量达到国家二级标准；城市供水、供热、燃气普及率分别达到85%、82%、75%，比2003年分别提高7.4个百分点、15个百分点和9.4个百分点。四年新建公路4980公里，公路里程达到8259公里，824个行政村通油（水泥）路。

　　——人民生活水平稳步提高。2007年城镇居民家庭人均可支配收入、农民人均纯收入分别达到11200元和2906元，年均分别增长14.9%和3.6%。大力拓展就业再就业渠道，开发公益性岗位8027个，年均新增就业4万人，城镇登记失业率控制在3.85%以下。加大养老、失业、医疗、工伤、生育保险扩面、基金征缴力度，保险金发放率100%。加强城市扶困和农村扶贫工作，符合条件的11.6万农村特困人口和8万城市特困居民全部纳入低保范围。

　　——社会建设全面发展。推进科技和人才工作，四年实施科技发展项目324项，引进各类人才1749人。基础和职业教育有了新发展，2007年高考本科及重点大学进线率、名牌大学录取率均创历史最好水平，白城卫校升格为高等专科学校，组建了白城职业技术学院。文化体育事业健康发展，成功承办了吉林省第三届二人转·戏剧小品艺术节暨白城文化旅游节等活动，竞技和群众性体育蓬勃发展。公共卫生体系不断完善。人口和计划生育工作完成了省下达计划。军民共建实现了全国双拥模范城“二连冠”。圆满完成了全国第二次农业普查任务。地方志工作受到国家表彰。民族、宗教、外事侨务、广播电视、老龄、妇女儿童、残联、红十字会等事业有了新发展，国防后备力量、人防、防震减灾等工作取得了新成绩。

　　——民主和法制建设不断深入。定期向人大报告、向政协通报工作，自觉接受人大和政协监督，四年办理人大代表建议202件、政协委员提案和建议案223件。深入开展普法和依法治市工作。加强信访工作。加大专项整治力度，安全生产形势基本稳定。强化社会治安综合治理，建立了治安巡逻防控机制，市区治安秩序进入历史最好时期。

　　——政府自身建设进一步加强。推进政府管理创新，开展了 “四服务”活动，优化了经济发展软环境。强化政务公开工作，实施了行政审批和收费集中办理，四年清理取消行政许可项目97项。市长公开电话群众投诉办结率和满意率分别达98.8%和96.7%。应急管理和预案体系进一步完善。治理商业贿赂、纠正部门行业不正之风和执法监察工作取得成效。

　　过去四年，我们坚持以发展为第一要务，树立科学发展观，推进了经济社会又好又快发展；坚持以工业园区和项目建设为突破口，推进经济总量和规模的扩张，增强了综合经济实力；坚持走改革开放之路，打开对外窗口，实现了白城的新发展；坚持以人为本，关注民生，认真解决群众最关心、最直接、最现实的问题，维护了群众利益；坚持在市委的正确领导下，团结一致、求实务实，形成了加快发展的强大合力。

　　四年来取得的成绩，是全市广大干部群众解放思想、开拓进取、团结奋斗的结果；是社会各界、各方面大力支持的结果。我代表市政府向全市工人、农民、知识分子、各阶层群众和各级干部，向各级人大、政协、民主党派、工商联、人民团体及各界人士，向驻白部队、武警官兵、公安司法干警和中省直驻白单位，向对我们工作给予关心和帮助的离退休老领导、老同志表示衷心的感谢和崇高的敬意！

　　同时，我们也清醒地看到发展中存在的问题，主要是：经济总量小、财政收入少、经济自主增长机制不强和投融资能力弱的状况尚未从根本上改变；工业规模小，城市化进程慢，农业产业化水平不高；科技创新能力不强，城乡居民收入水平低，就业和社会保障压力大，社会不稳定因素仍然存在等。针对这些困难和问题，新一届政府将竭尽全力，对症施策，以对历史和未来高度负责的精神，解决发展障碍问题，力争实现新的更大的突破。

　　二、今后五年的目标和任务

　　未来五年是白城步入投资强力拉动、项目全面推进、产业迅速壮大、经济高效增长、总量快速集聚的关键时期，我们要抓住机遇，积蓄力量，发挥优势，乘势而上，全力推进白城的加速发展和全面振兴。到2012年，全市地区生产总值完成530亿元，年均增长18.4%；固定资产投资完成500亿元，年均增长20.1%；全口径财政收入30亿元，年均增长11.9%；城镇居民人均可支配收入18500元，年均增长10.6%；农民人均纯收入4500元，年均增长9.1%；人口自然增长率控制在4.5‰以内。人居条件和生存环境不断优化；民主法制建设步伐加快；群众科学文化、思想道德、健康素质全面提高；生态环境得到改善，可持续发展能力进一步增强，综合实力明显提升。

　　实现未来五年的发展目标，市政府要高举邓小平理论和“三个代表”重要思想伟大旗帜，全面贯彻落实党的十七大精神，以科学发展观统领全局，继续解放思想，坚持改革开放，推动科学发展，促进社会和谐，按照市第四次党代会确定的指导思想和工作方针，坚持一切从实际出发，实事求是，充分认识和把握市情，在着力培育六大产业的同时，围绕能源产业兴市，加快打造火电之城、风电之城、石油之城；围绕特色农业裕民，加快建设水稻基地、燕麦基地、杂粮杂豆油料基地；围绕生态产业利国，加快建设草原湿地之乡、林木瓜果之乡、绿色产品之乡。求真务实，埋头苦干，努力提高我市综合经济实力、和谐社会建设水平和人民生活质量，加快白城经济崛起、社会和谐、人民富裕进程。

　　今后五年政府工作的主要任务：

　　（一）突出园区带动和投资拉动，强力推进项目建设，提升经济发展速度和质量。发挥全市工业园区项目集聚和产业带动作用，进一步完善基础、优化环境，搞好产业布局，加快产业集群式发展，把园区建成重大项目孵化基地、产业化龙头企业基地、高新技术产业培育基地、新型工业化示范基地，成为重要的经济增长点和迅速壮大的工业新城。

　　围绕能源产业兴市，积极运作实施火电、风电、石油开发项目，加快打造火电之城、风电之城、石油之城。组织实施500万千瓦火电、300万千瓦风电、200万吨石油建设项目。火电建设在着力推进白城电厂2×60万千瓦火电新建、白城热电厂扩建以及500千伏输变电工程建设的同时，做好通榆500千伏输变电等工程的前期工作，积极运作大安、镇赉等火电项目，以火电规模的不断壮大，加快打造火电之城；风电开发在加快运作国家和省已批准风电项目的同时，积极实施通榆40万千瓦国家特许经营权项目，着力推进通榆、洮南向阳两个百万千瓦和镇赉建平等风电场建设。统筹风力资源，搞好风电发展规划，严格准入标准。积极引进和发展风电设备制造业及相关产业，以风电规模的不断扩张，加快打造风电之城；充分发挥我市石油蕴藏丰富的优势，加快大安红岗子、美威油田开发，建成镇赉英台年100万吨采油场，搞好套保油田试验，积极发展以石油、天然气为原料的化工产业，以石油产业的崛起，加快打造石油之城。同时，充分利用内蒙古霍林河、伊敏河以及黑龙江和我市的煤炭资源，在发展火电的同时，积极谋划发展煤化工产业，搞好煤矸石、粉煤灰的综合利用，培育新的优势产业。

　　围绕特色农业裕民，积极运作实施水利和农业产业化项目，加快建设水稻基地、燕麦基地、杂粮杂豆油料基地。全力推进引嫩入白、大安灌区、镇赉土地整理开发等项目建设，加快运作和启动大安土地整理开发、引洮入向、人工降雨基地二期等工程，积极争取国家及亚行对洮儿河灌区工程的投资，科学利用江河水、洪水和空中云水资源，在全面提高水利保障能力的同时，大力实施水田开发，到2012年，全市水田面积发展到300万亩以上，水稻产量达到30亿斤，成为全省最具规模和实力的水稻基地；发挥我市燕麦科研、良种繁育、产品加工等优势，继续扩大种植规模，到2012年，燕麦种植面积达到20万亩以上，产量达到6000万斤以上，把白城建设成为国家级燕麦科研、良种繁育、示范推广和现代化产业技术体系创新基地；积极运作实施杂粮杂豆油料产业化项目，继续扩大杂粮杂豆油料种植规模，形成新的发展优势，把我市建设成为吉林西部的杂粮杂豆油料基地。大力发展以水稻、燕麦、杂粮杂豆油料、奶牛、肉牛、肉羊等为重点的特色农畜产品加工业，培育名牌产品，做大做强农特产资源加工产业，加快农业产业化和农民增收致富步伐。

　　围绕生态产业利国，积极运作实施绿色生态产业项目，着力打造草原湿地之乡、林木瓜果之乡、绿色产品之乡。坚持经济效益、社会效益和生态效益并重，继续实施“三北四期”、“退耕还林还草”和盐碱地、沙丘综合治理工程，在加快建设100万亩瀚海桑田、150万亩山杏和200万亩白刺三大生态产业基地的同时，发挥我市草原湿地、林地广阔和绿色生态产品丰富的优势，以引嫩入白等水利工程为保障，搞好草原湿地的恢复和保护，有计划地发展生态和经济价值兼备的适地植被，以良好的生态，打造名副其实的草原湿地之乡；围绕山杏、白刺、黑水西瓜、南国梨等具有地方特色的林木瓜果优势，积极运作实施产业开发项目，推进瓜果生产由“一乡一业”向“数乡一业”转变，形成产业规模，以葱郁的经济林木和飘香的瓜果，打造林木瓜果之乡；依托无污染的生态环境和龙头企业，强化农产品质量安全体系和无规定动物疫病区建设，积极扩大标准化无公害、绿色、有机农畜和水产品生产，大力开发和生产绿色营养品、保健品，着力发展缫丝、丝绸及加工产业，以绿色优生态，变绿色为财富，以自然健康的特色精品，打造绿色产品之乡。

　　在加快能源、特色农业和生态项目建设的同时，发挥通道对经济发展的助推作用，协调搞好霍林河至白城运煤通道建设；加快运作白城机场前期工作，力争2009年开工建设；积极争取实施松原—白城—石头井子高速公路、嫩江大桥等项目，提高我市交通设施水平。

　　（二）围绕经济发展的重点领域，调整优化产业结构，继续推进经济稳步持续快速发展。壮大支柱和优势产业，推进工业化进程。积极探索走新型工业化道路，以信息化改造提升传统产业。坚持以市场为导向、企业为主体、工业园区为重要载体，增强自主创新能力，发展循环经济。着力推进以能源、特色农产品加工、汽车零部件、纺织服装、医药、建材为主体的支柱和优势产业发展，加快技术进步，争创名牌产品，大力培育发展新兴和劳动密集型产业，以工业的率先崛起，带动我市经济加快发展。

　　提升农业产业化水平，建设社会主义新农村。根据白城实际，大力发展适应农业、避灾农业、市场农业和效益农业。在加快建设水稻、燕麦、杂粮杂豆油料基地的同时，积极培育龙头企业，组织实施200万吨玉米加工、100万吨水稻加工、50万吨葵花加工、20万吨辣椒加工、10万吨绿豆加工、10万吨蓖麻加工、10万吨汉麻加工、7万吨黑大豆加工、3万吨燕麦加工等系列农产品加工项目。着力抓好50万吨鲜奶加工、10万头肉牛加工、100万只肉羊加工、1000万只白鹅加工、100万只狐貉加工等养殖加工一体化项目建设。发展农村新型合作组织，建立信息服务体系，实现产业、城乡和区域联动，提高农业市场化、组织化程度。推进新农村建设，发展农村经济，增加农民收入，加快脱贫致富，改善农村面貌。

　　积极发展现代服务业，提高服务业比重和水平。大力发展面向生产的服务业，优先发展现代物流业，加快发展金融业，鼓励发展商务服务业，支持发展文化创意产业。规范提升面向生活的服务业，在推进连锁、特许经营、物流配送等现代流通方式的同时，加大旅游开发力度，全力打造“草原风光、湿地风貌、民族风情、风电景观”特色旅游品牌。

　　（三）进一步深化改革扩大开放，加快体制机制创新，借助外力实现白城的新发展。抓好重点领域改革，增强经济发展活力。围绕解决影响发展的深层矛盾和问题，继续推进政企、政事、政资分开。进一步深化国有企业改革，加快民营化步伐，建立现代企业制度。鼓励引导和扶持民营经济加快发展，努力创造宽松、公平的竞争环境，重点培育科技型、外向型、劳动密集型民营企业。围绕新农村建设，统筹抓好农村综合配套改革。深化行政管理体制和事业单位人事制度改革，严格人员编制管理，禁止超编进人。同时，做好其它各项改革工作，增强发展活力。

　　加大对外开放力度，借助外力实现发展。发挥区位、资源等比较优势，继续坚持“走出去、请进来”战略，充分利用国际国内两个市场、两种资源，进一步扩大开放领域、优化开放结构、提高开放质量，建立全面开放型经济发展格局。抓好招商引资工作，推进招商思路、方式和制度创新，积极开展各类经贸洽谈和项目推介活动。加快培育多元化出口主体，着力发展外向型企业、开发外向型产品，扩大出口规模，提高利用外资水平。充分发挥开发区对外开放的载体和窗口作用，大力引进资金、人才、技术和先进的管理经验，加快壮大开发区经济。

　　（四）立足市情和财力，继续实施民生民利工程，全面提升城市建设管理水平。围绕打造区域性中心城市，根据市情和财力，走向清洁要市容、向规划要形象、向绿化要特色、向环保要水平之路，推进城市建设管理市场化、规范化，努力把我市建成一座干干净净、整整齐齐、郁郁葱葱、亮亮堂堂，充满生机活力的草原新城。

　　加快基础设施建设，提升城市功能。五年计划投融资120亿元，继续实施民生民利工程建设。围绕改善人居条件，重点抓好棚户区及危旧房改造工程，积极推进旧住宅区综合整治；围绕完善城市功能，加快供热管网改造、市中心医院、特殊教育学校、城市污水、医疗废弃物、生活垃圾处理等工程建设；围绕改善交通条件，积极规划实施城市道路、城郊次干道及巷道、金辉互通立交桥等工程建设；围绕提高规划水平，进一步调整优化城乡布局；围绕美化城市环境，继续抓好城市园林绿化、广场、环城河等工程建设，进一步改善城市面貌。

　　加强城市规划和管理，提高管理水平。强化规划意识，树立规划权威。积极引进市场机制，推进城市管理体制改革。强化城管综合执法工作，搞好城市卫生和污染治理，建设环境友好型城市。

　　（五）坚持以造福于民为己任，推进以改善民生为重点的社会建设，营造和谐稳定的发展环境。大力开展创业促就业活动，推行灵活多样的就业方式，创造更多的就业机会。加强社会保障体系建设，积极构建“养老、失业、医疗、工伤、生育”五位一体的社会保险制度。搞好城市扶困和农村扶贫开发，加快社会福利、残疾人和慈善事业发展。做好住房公积金工作，健全廉租房制度，推动住房消费。加强国土资源管理，强化耕地特别是基本农田保护。建立完善卫生医疗保障体系，发展社区卫生服务和新型农村合作医疗事业。

　　推进平安白城建设。坚持归口办理、分级负责、属地管理，妥善处理信访问题，解决群众困难，化解社会矛盾。加强社会治安综合治理，依法打击各类违法犯罪活动。强化安全生产监督和管理，防止重特大事故的发生。推进民主法制建设，自觉接受人大依法监督和政协民主监督。加大从源头上防治腐败力度，促进廉洁从政。搞好“五五”、“六五”普法工作，推进依法治市和依法行政。

　　发展各项社会事业。加快科技进步和创新，增强科技对经济发展的支撑能力。坚持教育优先发展方针，巩固义务教育，发展职业教育，普及高中阶段教育，办好高等教育。加大人才培育和引进力度。强化精神文明建设。做好人口和计划生育工作。推进广播电视、体育、卫生等各项事业健康发展。

　　三、2008年重点工作

2008年是新一届政府的开局之年，我们要深入贯彻落实党的十七大精神，按照省委、省政府和市委四届二次全会的部署，坚持以发展为第一要务，抢抓机遇，奋力拼搏，强力推进投资拉动、项目促动、改革推动、开放带动，着力惠及民生、维护民利，发展各项社会事业，提高群众生活水平，加快白城发展步伐。

　　主要预期目标：地区生产总值完成276亿元，同比增长21%；固定资产投资完成240亿元，同比增长20%；全口径财政收入18.5亿元，同比增长8.4%；城镇居民人均可支配收入12320元，农民人均纯收入3197元，同比均增长10%。

　　完成上述目标，重点抓好以下工作：

　　（一）以工业园区建设为核心，培育壮大工业新城。把工业园区作为工业项目、产业发展的主体功能区和增量集聚的核心区，认真落实项目招商责任制，大力引进投资规模大、技术含量高、市场前景好、发展潜力大的工业项目。搞好园区产业布局，形成大中小一起发展、上下游产业协调推进的发展格局。继续完善各项基础设施，提高服务质量，加快打造以园区为核心，带动其它相关产业共同发展的工业新城。

　　坚持新增项目和产业扩能结合、对外招商与对内服务并重、培植远期税源和壮大当期税源兼顾，围绕企业优质资产，加大重点企业招商重组力度，使企业成为市场的主体。加快实施一批技术先进、附加值高、市场竞争力强的技改项目，增强企业发展后劲。组织好提速增效工作，通过强化管理、搞好服务，激发企业活力。坚持不懈地抓好节能降耗和污染减排工作。继续实施名牌带动战略。全市规模以上工业增加值和利润增幅分别达到16.5%和18.5%以上。

　　（二）以抓投资上项目为重点，加快能源基地建设。2008年，全市规划千万元以上重点项目206个，计划投资120亿元。其中市直102个，计划投资60亿元。

　　随着风电项目的快速推进，火电项目的奠基，白城能源产业异军突起，以火电、风电、石油和煤炭开发为重点的能源基地建设已成为白城的发展方向。大力推进白城电厂2×60万千瓦火电新建、白城热电厂扩建工程建设，完成土建基础和部分设备购置等阶段性任务。500千伏输变电项目争取明年5月前开工。镇赉建平、洮南同力风电续建项目，洮北华能二期、宝山一期、双龙一期风电新建项目，力争年内并网发电；完成通榆、洮南向阳两个百万千瓦风电和通榆40万千瓦风电国家特许经营权项目的当年装机任务；抓紧做好白城火电新建二期、镇赉火电、大安火电及中广核风电二期等项目的前期工作；推进镇赉、大安石油勘探和开发，积极谋划石油和煤炭化工项目，以能源项目壮大能源产业，以能源产业构建能源基地。

　　在工业项目上，加快洮南名门塔筒扩建、天奇新能源设备制造项目建设，力争年内竣工投产；完成中小企业创业园标准厂房建设、吉林康熹保健产品GMP认证、镇赉银诺克GMP改造；推进洮北宏帝达重型卡车变速箱、鑫宇汽车线束项目建设，确保年内试生产；完成金升镍业、鑫鹏齿轮重型变速箱总成等项目的阶段性任务。

　　在农产品加工项目上，积极推进镇赉绿禾10万吨玉米功能糖、大安明胶扩产改造、洮北哈工大日处理500吨鲜奶、美利信汉麻加工、公益源有机黑大豆综合加工、尼龙1010、燕麦饮品等项目建设，确保年内建成投产；加快镇赉飞鹤乳制品加工、金利源食品、深圳工业园等项目建设。积极谋划特色农业和生态产业项目。

　　在基础设施项目上，引嫩入白工程力争完成五家子泵站建设，确保总干渠明年5月1日通水；大安灌区、镇赉土地整理开发等工程完成阶段性任务；启动引洮入向和以月亮湖蓄滞洪区为重点的洪水资源化利用等工程；推进大安土地整理开发、嫩江堤防汉书闸等工程，确保尽早开工。同时，积极做好白城机场、人工降雨基地二期、松原—白城—石头井子高速公路、工业园区铁路专用线等项目的前期工作。

　　（三）以新农村建设为契机，全力推进农业产业化。走特色农业裕民之路，调整优化结构，在扩大水稻、燕麦、杂粮杂豆等种植面积，稳步提高粮食综合生产能力的同时，继续扩大辣椒、蓖麻、葵花、芝麻、瓜果等特色农产品规模。围绕发展精品牧业，抓好优势农畜产品基地建设，壮大奶牛、肉牛、肉羊、白鹅、水产和狐貉养殖等特色产业。搞好动植物疫病防控，强化农畜产品质量安全管理。培育农村新型合作组织，支持产业化经营和龙头企业发展。鼓励农村劳务输出，增加农民收入的工资性比例。

　　加强农业基础设施建设。加快制定实施全市《水利发展五年规划》，在全力抓好引嫩入白、大安和洮儿河灌区等水利工程建设的同时，继续搞好江河堤防、水库除险加固、抗旱水源、农村饮水安全等工程建设，做好人工增雨工作，提高抵御自然灾害的能力。同时，加快推进瀚海桑田、山杏、白刺三大生态产业和草原湿地、林木瓜果、绿色产品基地建设。

　　组织好新农村建设试点工作。在发展农业生产的同时，加快农村道路、电力、通讯、医疗、学校、广播电视等基础设施建设，推进“泥草房”改造，改善农民生产生活条件。

　　（四）以现代服务业为方向，进一步繁荣商贸经济。发挥区位优势，着力发展有特色、成规模、辐射力强的专业市场和要素市场。在巩固传统服务业的同时，大力发展金融保险、商务服务、信息中介等现代服务业，积极承接国内外发达地区服务贸易转移，发展连锁、特许经营和物流配送等现代流通方式，提升服务业整体水平。全市社会消费品零售总额增长10%。积极发展对外贸易，增强出口创汇和利用外资能力，外贸进出口总额增长8.3%。

　　大力发展旅游产业。认真实施全市旅游发展总体规划，强化基础设施建设，积极挖掘向海、莫莫格等重点景区景点生态和文化内涵，开发特色旅游项目和旅游产品，拓展旅游市场，全年接待国内外游客和旅游收入同比分别增长13%和10%。

　　（五）以推进改革开放为手段，增强经济发展活力。巩固深化国企改革成果，建立符合市场经济要求的运营机制。抓好国有厂办大集体企业、集体林权、水管体制改革，统筹搞好农村综合改革，加快小城镇改革，配套推进财税、投资、金融体制及生产要素市场改革，切实抓好其它领域的改革。

　　强化招商引资和对外开放工作。落实领导包保、部门主抓、具体人员负责制度，围绕全市各工业园区、开发区以及六大产业和特色资源，搞好项目包装，面向国内外发达地区开展招商活动，努力扩大招商成果。全年引进域外资金同比增长15%。发展壮大民营经济。继续实施民营经济腾飞计划，大力推进全民创业。认真落实支持民营经济发展的各项政策，扶持和帮助民营企业扩大规模、提高水平，使民营经济成为推动我市经济发展的重要力量。

　　（六）以民生民利工程为基础，着力提升城市品位。继续实施民生民利工程，坚持向地下用力、向平房倾斜、向改革求活力、向管理要效益，量力而行、尽力而为，努力打造居安、行畅、天蓝、地绿、水美之城。市区计划投融资21.2亿元，努力办好10件实事。

　　一是对27个小区115.9万平方米的棚户区及危房进行改造，建设6个标准化小区，使8400户棚户区及危房居民乔迁新居。二是对48.8万平方米的16条街路、64条城郊次干道及巷道进行维修铺设，解决5万城郊居民出行难问题。三是开展水源地保护工程的征地、圈围栏和垃圾填埋工作，保障城市居民饮用水安全。四是完成污水处理厂一期续建工程建设，启动实施二期工程，提升污水处理能力。五是启动中医院改造、市生殖保健医院等工程建设，提高公益设施水平。六是实施白城热电厂供热管网改造配套工程的锅炉安装及部分管网改造，使市区集中供热率提高到85%。七是对25条街路、8处绿地、10条街路分车岛以及公园、广场等进行绿化，提高绿化档次和品位。八是实施市区转盘广场改造工程，进一步优化城市形象。九是搞好市区路灯维护和灯型调试，使临街商家亮灯率达到98%以上。十是开展市容市貌及交通秩序综合治理，继续对各类非法营运车辆进行整治，提高城市管理水平。

　　（七）以建设和谐白城为目标，发展各项社会事业。突出抓好就业、社会保障和救助体系建设，多渠道开发就业岗位，城镇新增就业37万人。完善养老、失业、医疗、工伤、生育保险制度，启动城镇居民医疗保险，做好城市扶困、农村扶贫、城乡低保和救灾救济工作。认真落实信访责任制，各级领导都要敢于、善于、积极、自觉接待群众上访，主动、及时、就地、依法解决群众反映的问题。做好市长公开电话工作。加大对重点行业、部位的监控整治力度，确保生产安全。加强治安防控体系建设，严厉打击各种违法犯罪活动，推进“平安白城”建设。

　　广泛开展社区、企业、乡村和谐创建活动，建设和谐文明城市。推进科技进步和创新，提高科技贡献率。整合教育资源，搞好农村中小学标准化建设，做好实施城市义务教育免除学杂费的准备工作。大力发展文化体育事业。加强公共卫生、疾病防控体系建设，提高城乡医疗水平。抓好人口和计划生育工作，统筹解决人口问题。做好国防后备力量、双拥共建工作，加强国防教育，完善动员机制，维护国防工程。做好广播电视、民族、宗教、外事侨务、防震减灾、气象、人工降雨、人防、地方志、老龄、妇女儿童、残联、红十字会等工作，加快社会事业发展。

　　四、加强政府自身建设把一切从实际出发，实事求是，求真务实，真抓实干作为本届政府的执政理念，坚持把握市情，准确定位，尊重规律，发挥优势，和谐发展，努力建设法治、服务、责任、效能型政府。

　　（一）坚持从白城实际出发，认真履行政府职责。深入贯彻落实党的十七大精神，以科学发展观为指导，在充分认识和把握市情的基础上，克服总量小、基础差等不利因素，充分发挥比较优势，准确定位，科学谋划，重实干、谋实策、说实话、办实事，确保政府各项工作和决策不偏离发展实际，少花成本，少走弯路，一步一个脚印地向前迈进。进一步强化政府职责，时刻把工作重心敲在求实务实上，做一切工作、办一切事情，都要下真功夫、出真力气、见真成效，走出一条切合白城实际的科学、高效、快速发展之路。

　　（二）坚持按客观规律办事，促进政府决策科学化。把坚持按客观规律办事作为推进工作、谋划发展的根本依据。一方面充分认识和把握规律，认真研究经济社会发展的宏观走势和微观变化，及时发现和把握倾向性问题，突出决策的前瞻性、务实性，防止规划失实和决策失误，科学制定发展战略。另一方面积极利用规律，在搞好调查研究的基础上，统筹兼顾眼前利益和长远利益，因势利导、趋利避害，提高决策的准确性，避免工作的盲目性。继续完善社情民意反映制度、社会听证制度和专家咨询制度，集中全民智慧，确保政府决策民主化、科学化。

　　（三）坚持推进依法行政，加快法治型政府建设。坚持向人大报告和政协通报工作制度，认真办理人大代表建议和政协委员提案，密切同各民主党派、工商联和人民团体的联系。继续推进政务公开，充分发挥各级政务大厅和行政审批服务中心的作用，减少审批事项，简化办事程序。认真贯彻执行《宪法》、《行政许可法》、《公务员法》、《依法行政实施纲要》等法律法规，严格规范性文件的制定和管理，继续搞好“五五”普法和行政复议工作，提高政府依法行政水平。

　　（四）坚持转变工作作风，全面提高政府行政效能。切实肩负起加快白城发展的历史重任，进一步增强工作的责任感和紧迫感，谋划力求“真”、工作力求“实”、发展力求“好”。切实改进学风和文风，精简会议和文件，坚决反对形式主义、官僚主义、弄虚作假。建立健全工作绩效评估和行政问责制，推进政务信息化建设，提高工作效率。倡导雷厉风行的工作作风，进一步强化“快”的意识、加大“跑”的步伐、树立“争”的勇气，脚踏实地，埋头苦干，争做一流工作，争创一流业绩。

　　（五）坚持强化廉政建设，树立人民政府的良好形象。认真落实党风廉政建设责任制，严格执行领导干部廉洁自律的各项规定。大力倡导勤俭节约、艰苦奋斗，反对奢侈浪费，降低行政成本，建设廉洁政府和廉价政府。强化软环境整治，努力营造良好的服务环境、规范的政务环境和诚信的市场环境。坚持标本兼治、综合治理、惩防并举、注重预防，逐步构建拒腐防变的长效机制。加大执法监察力度，坚决纠正损害群众利益的突出问题。切实落实“一岗双责”目标责任制。

　　各位代表，本届政府的任务目标已经明确，使命光荣、责任重大、发展充满希望。我们要以党的十七大精神为动力，在市委的正确领导下，在市人大、市政协的监督支持下，以求实务实的精神，开拓进取的工作，奋发图强的意志，努力开创各项工作的新局面，为实现白城经济崛起、社会和谐、人民富裕而努力奋斗！
